# May-Thurner Syndrome Is Aggravated by Pregnancy

**DOI:** 10.3390/medicina57030222

**Published:** 2021-03-01

**Authors:** Kuntharee Traisrisilp, Manatsawee Manopunya, Tanop Srisuwan, Wisit Chankhunaphas, Theera Tongsong

**Affiliations:** Department of Obstetrics and Gynecology, Faculty of Medicine, Chiang Mai University, Chiang Mai 50200, Thailand; kuntharee.t@cmu.ac.th (K.T.); manatsawee.m@cmu.ac.th (M.M.); tanop.s@cmu.ac.th (T.S.); wisit.chank@gmail.com (W.C.)

**Keywords:** deep vein thrombosis, May-Thurner syndrome, pregnancy

## Abstract

This study aims to emphasize that asymptomatic patients with undiagnosed and asymptomatic May-Thurner syndrome (MTS) may firstly develop severe compression during pregnancy. A 40-year-old woman, G1P0, at 22 weeks of twin gestation presented with left lower extremity edema and pain. One twin was structurally normal while the other had bilateral renal agenesis with oligohydramnios. Magnetic resonance venography (MRV) revealed severe compression of the left iliac vein by the right iliac artery without evidence of deep vein thrombosis (DVT). Conservative treatment with anticoagulant prophylaxis was instituted throughout the rest of pregnancy and postpartum period. She was also complicated with severe pre-eclampsia, a cesarean section was performed due to a prolapsed cord at 27 weeks of gestation, and she gave birth to a surviving baby weighing 1100 g. In conclusion, this case report provides evidence that pregnancy can disclose a subtle May-Thurner anatomy to be symptomatic without DVT. Successful pregnancy outcomes could be achieved with conservative treatment and anticoagulant prophylaxis.

## 1. Introduction

May-Thurner syndrome (MTS) is an iliac vein compression syndrome, first described by May and Thurner in 1957 [1]. It is a condition involving the left common iliac vein being compressed by the right common iliac artery against the fifth-lumbar vertebra. This anatomical variant can lead to obstruction of venous drainage from the left lower extremity, resulting in an increased risk of left-sided deep venous thrombosis (DVT), or lymphedema. The pregnancy and postpartum period is a life-period that increases the risk of thromboembolism. Therefore, DVT among patients with MTS may be aggravated by pregnancy or postpartum. Nevertheless, most patients with this syndrome have no clinical symptoms throughout their lives. Thus, some authors prefer using the term “May-Thurner anatomy” for asymptomatic patients and the term “May-Thurner syndrome” for those with clinical symptoms/signs of venous obstruction [2]. MTS associated with DVT during pregnancy and postpartum has been reported in a very limited number of studies [3,4,5,6], mostly in postpartum. In this report, we described a unique case of MTS with the first appearance of left leg edema during pregnancy in a patient who had had no clinical manifestations of MTS before. The main objective of this report is to emphasize that asymptomatic patients with undiagnosed May-Thurner anatomy may first develop clinical symptoms and signs during pregnancy.

## 2. Case

**Figure 1:**
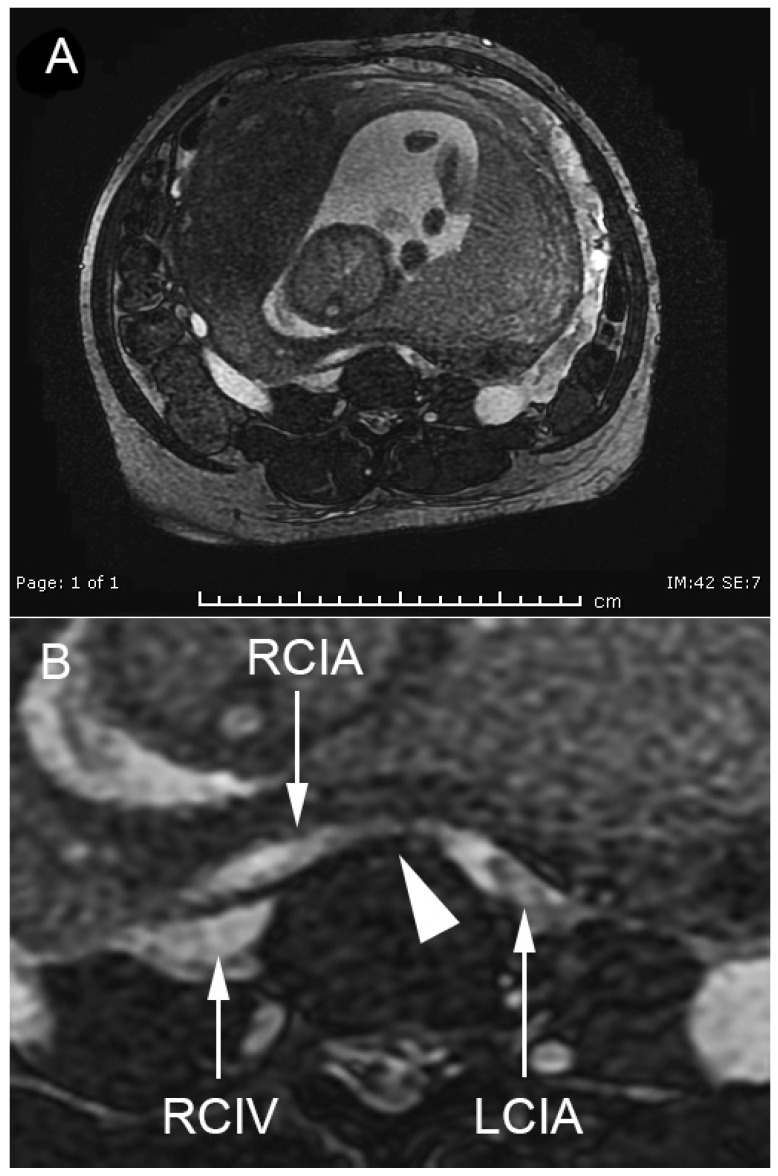
Magnetic resonance venography (MRV), the cross-section of the pelvis shows severe compression of the left iliac vein by the right iliac artery at 22 weeks of gestation. (LCIA: left common iliac artery; RCIA: right common iliac artery; RCIV: right common iliac vein; arrowhead indicating complete obstruction of the left common iliac vein by RCIA). (**A**), overview of the whole pelvis; (**B**), magnification of the area of May-Thurner point.

**Figure 2:**
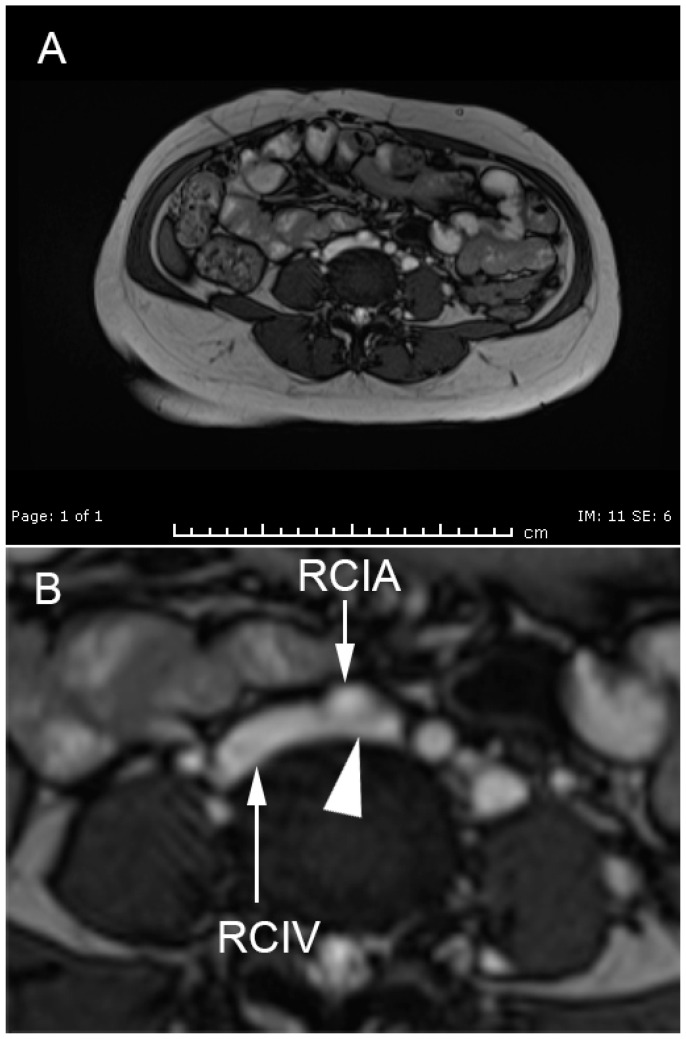
MRV shows partial compression at the May-Thurner point at 2 months after birth. (RCIA: right common iliac artery; RCIV: right common iliac vein; arrowhead indicating partial compression of the left common iliac vein by RCIA). (**A**), overview of the whole pelvis; (**B**), magnification of the area of May-Thurner point.

A 40-year-old woman, G1P0, at 22 weeks of gestation presented with left lower extremity edema and pain for one week prior to admission. She had primary infertility caused by bilateral endometriomas and history of operations of right salpingo-oophorectomy and left ovarian cystectomy. The current pregnancy was an IVF (in vitro fertilization) conception with egg donation and 2-blastocysts transfer, resulting in the twin gestation. An ultrasound examination at 7 weeks of gestation revealed twin pregnancy with a dichorionic-diamnionic (DCDA) placenta, and the fetal biometry was consistent with the menstrual age. Furthermore, she had chronic hypertension that was well-controlled with medication (methyldopa). Additionally, she had intramural leiomyoma at the right antero-lateral uterine wall (approximately 8 cm diameter). However, until this admission, her course of pregnancy was uneventful. She had had no history of thromboembolism or any symptoms and signs of venous thrombosis before. On physical examination, the left lower extremity was obviously edematous, compared to the right one, but had no inflammatory signs. All other organs, as well as the uterus, were within normal limits. All the basic laboratory findings for standard antenatal care revealed normal results. An ultrasound screening for anomaly at mid-pregnancy showed bilateral renal agenesis with severe oligohydramnios in one twin, whereas the other twin was structurally normal with normal growth and the amniotic fluid volume was normal. Deep vein thrombosis (DVT) was highly suspected. However, compression ultrasonography of the left lower extremity revealed a normal result (no evidence of thrombosis), but the vessels in the left lower extremity were generally dilated. Magnetic resonance angiography (MRA) and magnetic resonance venography (MRV) (axial FIESTA) of the pelvic cavity and lower inferior vena cava (IVC) showed severe compression of the left common iliac vein (Figure 1), in both the supine and right lateral decubitus positions, and severe compression of the left external iliac vein, but with an improvement of patency in the right lateral decubitus position. No evidence of deep vein thrombosis was observed. The right common and external iliac vein and right femoral vein were patent. May-Thurner syndrome was diagnosed. Enoxaparin (40 mg subcutaneously administered daily until 6 weeks postpartum) for DVT prophylaxis was prescribed. Conservative and symptomatic treatment of leg pain was given as needed. The pregnancy was continued, aimed at term. However, she developed superimposed pre-eclampsia at 23 weeks of gestation and was admitted and conservatively treated throughout the rest of the pregnancy. Moreover, she developed severe pre-eclampsia at 26 weeks of gestation, and an antihypertensive drug (hydralazine), anticonvulsant (magnesium sulfate), and dexamethasone for fetal lung maturity were given. At 27 weeks of gestation, conservative treatment was stopped because of its failure to control blood pressure and the occurrence of spontaneous labor. The anticoagulation was also discontinued. During labor, cord prolapse occurred and an emergency cesarean section was performed. The normal preterm twin was delivered weighing 1100 g, with 1-, 5-, and 10-min Apgar scores of 2, 6, and 8, respectively. The twin with bilateral renal agenesis died shortly after birth. The normal baby survived with normal development and was discharged at 2 months of life. The patient underwent an MRV at 2 months postpartum, showing May-Thurner anatomy with partial obstruction of the left common iliac vein by the right common iliac artery, and no deep vein thrombosis (Figure 2). The patient had no DVT, no leg edema, and she needed no further anticoagulation.

## 3. Discussion

The case presented here is a unique case of MTS. It is an important addition to the existing cases in the literature in terms of severe compression of the iliac vein being induced by pregnancy without evidence of DVT, and the fact that the compression was spontaneously relieved by postpartum involution with the disappearance of left leg edema. Our case indicates that, at least in some cases, the severity of left extremity edema is not always caused by DVT associated with hypercoagulability that is secondary to pregnancy. This case illustrates that the clinical manifestation of MTS is not always attributed to DVT, but the compression itself could cause significant clinical symptoms. Therefore, this case is unique and different from previously reported cases [4,5,7,8] in which DVT was the main clinical problem. As is already known, pregnancy is a hypercoagulable state and significantly increases the risk of DVT, especially in cases at higher risk, like patients with MTS. This case also indicates that pregnancy can aggravate the severity of the compression apart from hypercoagulation and DVT. In other words, pregnancy can disclose previously subtle May-Thurner anatomy to be clinically severe, which becomes asymptomatic again after birth. However, obstruction with delayed venous return certainly increases the risk of DVT; therefore, this patient was given prophylactic anticoagulation throughout the rest of pregnancy and postpartum period. We postulated that hypervolemia, which is a physiologic change of pregnancy (an increase in blood volume by about 40%–50% and vessel size), and an enlarged gravid uterus may aggravate the subtle compression, making it symptomatic. Additionally, large uterine size, associated with uterine leiomyoma and twin pregnancy, in this case, might cause the compression at the May-Thurner point to be more severe.

We noted that the patient developed early-onset pre-eclampsia with severe features. We believe that MTS and pre-eclampsia in this case were a coincidental finding rather than a significant association. Based on the literature review of a very limited number of cases, the association between the two entities has never been reported. Rather, chronic hypertension was likely related to the development of pre-eclampsia in the case presented here. Clinically, leg edema in this case was unlikely caused by pre-eclampsia, which causes symmetrical edema in both legs, in contrast to the case in this study, which involved only the left leg.

Based on this case and previous case reports, we emphasize that MTS might be underdiagnosed due to the absence of symptoms and the fact that the condition is first recognized during pregnancy. The recognition of MTS may help to increase awareness of situations that increase the risk of DVT in the future, such as the use of oral contraceptive pills, having antiphospholipid syndrome, prolonged operations, etc. Pregnancy may not be contraindicated in women of reproductive age with this condition, and asymptomatic patients may not need anticoagulant prophylaxis, but close clinical and ultrasound follow-up is warranted during the pregnancy and postpartum period. Importantly, MTS predominantly affects women of reproductive age, with the preponderance of cases occurring during pregnancy or due to combined contraceptive use. Although MTS is relatively rare, the exact prevalence is underestimated, and it can be life-threatening because of the development of DVT with the serious potential consequences of pulmonary embolism [9], cerebral infarction caused by embolic phenomenon in the case of a patent foramen ovale [10], and acute limb ischemia with total intravenous thrombotic obstruction [4].

In conclusion, this case report provides evidence that pregnancy can further aggravate the compression of left iliac vein by the right iliac artery at the May-Thurner point and can cause marked edema on the left lower extremity with spontaneous resolution after birth. Pregnancy can disclose subtle May-Thurner anatomy by making it symptomatic for the first time. Successful pregnancy outcomes could be achieved with conservative treatment, and serious morbidity secondary to DVT can be effectively prevented with anticoagulant prophylaxis. This case is a lesson that isolated left leg edema during pregnancy should prompt the listing of MTS in differential diagnoses to achieve early detection before the development of DVT, enabling the provision of effective prophylaxis for serious complications.

Take-away lessons derived from this study are as follows:

- Pregnancy can disclose undiagnosed or absolutely asymptomatic MTS for the first time by aggravating the subtle obstruction to be clinically severe;
- In a patient with edema specific to the left lower extremity during pregnancy, MTS should be listed in differential diagnoses;
- In pregnancy, MTS with marked edema can be solely caused by obstruction at the May-Thurner point without DVT;
- MTS without DVT during pregnancy may need a prophylactic rather than therapeutic anticoagulant;
- The pregnancy-induced obstruction secondary to MTS is expected to spontaneously disappear after delivery.

## Data Availability

The data of this report are available from the corresponding authors upon request.

## Abbreviations

DVT	Deep vein thrombosis
DCDA	Dichorionic-diamnionic
MRV	Magnetic resonance angiography
MRV	Magnetic resonance venography
MTS	May-Thurner syndrome
